# Feasibility of titrating PEEP to minimum elastance for mechanically ventilated patients

**DOI:** 10.1186/s40814-015-0006-2

**Published:** 2015-03-21

**Authors:** Yeong Shiong Chiew, Christopher G Pretty, Geoffrey M Shaw, Yeong Woei Chiew, Bernard Lambermont, Thomas Desaive, J Geoffrey Chase

**Affiliations:** 1 Department of Mechanical Engineering, University of Canterbury, Private Bag, 8140, Christchurch, New Zealand; 2 Department of Intensive Care, Christchurch Hospital, Christchurch, New Zealand; 3 Western Medicine Division, Hospital Lam Hua EE, Pulau Penang, Malaysia; 4 GIGA Cardiovascular Science, University of Liege, Liege, Belgium

**Keywords:** ARDS, Respiratory elastance, Dynamic functional residual capacity, PEEP, Mechanical ventilation

## Abstract

**Background:**

Selecting positive end-expiratory pressure (PEEP) during mechanical ventilation is important, as it can influence disease progression and outcome of acute respiratory distress syndrome (ARDS) patients. However, there are no well-established methods for optimizing PEEP selection due to the heterogeneity of ARDS. This research investigates the viability of titrating PEEP to minimum elastance for mechanically ventilated ARDS patients.

**Methods:**

Ten mechanically ventilated ARDS patients from the Christchurch Hospital Intensive Care Unit were included in this study. Each patient underwent a stepwise PEEP recruitment manoeuvre. Airway pressure and flow data were recorded using a pneumotachometer. Patient-specific respiratory elastance (*E*_*rs*_) and dynamic functional residual capacity (dFRC) at each PEEP level were calculated and compared. Optimal PEEP for each patient was identified by finding the minima of the PEEP-*E*_*rs*_ profile.

**Results:**

Median *E*_*rs*_ and dFRC over all patients and PEEP values were 32.2 cmH_2_O/l [interquartile range (IQR) 25.0–45.9] and 0.42 l [IQR 0.11–0.87]. These wide ranges reflect patient heterogeneity and variable response to PEEP. The level of PEEP associated with minimum *E*_*rs*_ corresponds to a high change of functional residual capacity, representing the balance between recruitment and minimizing the risk of overdistension.

**Conclusions:**

Monitoring patient-specific *E*_*rs*_ can provide clinical insight to patient-specific condition and response to PEEP settings. The level of PEEP associated with *minimum*-*E*_*rs*_ can be identified for each patient using a stepwise PEEP recruitment manoeuvre. This ‘minimum elastance PEEP’ may represent a patient-specific optimal setting during mechanical ventilation.

**Trial registration:**

Australian New Zealand Clinical Trials Registry: ACTRN12611001179921.

**Electronic supplementary material:**

The online version of this article (doi:10.1186/s40814-015-0006-2) contains supplementary material, which is available to authorized users.

## Background

Mechanical ventilation (MV) is used in the intensive care unit (ICU) to support the breathing of patients with respiratory failure. MV has evolved from a supporting role to a therapy that affects disease progression and outcome [1-5]. Hence, it is important to have optimal MV management to support patient recovery [5-7].

Positive end-expiratory pressure (PEEP) is one of the important MV settings for patients with acute respiratory distress syndrome (ARDS) [8-10]. PEEP keeps alveoli open and maintains recruitment [11,12]. Several attempts have been made to standardize MV therapy, especially PEEP selection [13-17]. However, these approaches were generalized from large population studies and did not consider intra-patient variability and inter-patient heterogeneity of ARDS. Thus, there is currently no conclusive result on PEEP selection [7,18-21]. Without a standard method for setting patient-specific PEEP, clinicians rely on intuition, experience and/or methods based on consensus guidelines or cohort-based outcomes [22], leading to more variable care and outcomes.

Studies by Suter et al. [23,24] have suggested that patient-specific PEEP selected at maximum compliance (or minimum elastance, where elastance = 1/compliance) may be beneficial. Similarly, recent animal studies have shown that optimal PEEP can be titrated to minimum respiratory elastance [25-27]. In particular, Carvalho et al., Suarez-Sipmann et al. and Lambermont et al. [25-27] have all reported that pigs with ARDS had minimum elastance at a specific PEEP associated with maximum recruitment, higher functional residual capacity, higher oxygenation and without lung overdistension. While these findings are consistent, the application of this PEEP selection method is limited in clinical practice, and current standard of care remains largely based on clinical intuition or a generalized approach such as using the ARDSNet PEEP-FiO_2_ table [13-17,22]. Recently, Pintado et al. [28] conducted a randomized controlled trial (RCT) that demonstrated benefits from selecting PEEP at minimum elastance. However, the results of this trial failed to reach statistical significance, with *p* = 0.16 [28]. Thus, there is great interest in standardizing patient-specific PEEP selection using the minimum elastance-PEEP method.

In this study, the feasibility of setting PEEP, for mechanically ventilated, intubated ARDS patients, using a patient-specific minimum elastance (*E*_*rs*_) is investigated. Each patient included in this study underwent a recruitment manoeuvre (RM) with multiple PEEP changes to evaluate the patient-specific *E*_*rs*_-PEEP profile and locate the point of minimum *E*_*rs*_. Clinical implications and feasibility of titrating PEEP to minimum *E*_*rs*_ to guide and improve therapy are discussed.

## Methods

### Patients

The study was conducted in the Christchurch Hospital ICU (New Zealand). Ten patients diagnosed with ARDS using the 1998 consensus [9,29] were recruited to the study on the basis that they had the following conditions: (1) acute onset of respiratory failure, (2) PaO_2_/FiO_2_ (PF ratio) between 150 and 300 mmHg, (3) findings of bilateral infiltrates on chest radiograph, and (4) absence of left-sided heart failure. The exclusion criteria for the study were (1) patients who were likely to be discontinued from MV within 24 h, (2) age <16 years, (3) moribund and/or not expected to survive for greater than 72 h, and (4) patient with minimal sedation, where additional sedation may lead to prolonged MV.

Written informed consent was obtained from the patient/family members or relatives. These trials and the use of the data were approved by the New Zealand, South Island Regional Ethics Committee. The trial is registered with Australian New Zealand Clinical Trials Registry (ACTRN 12611001179921).

### Ventilator settings and measurements

All patients were ventilated using Puritan Bennett PB840 ventilators (Covidien, Boulder, CO, USA) with volume control (tidal volume, *V*_*t*_ = 6 ~ 8 ml/kg), synchronized intermittent mandatory ventilation (SIMV) mode. Other ventilator settings were not changed during the trial.

Airway pressure (*P*_*aw*_) and flow data (*Q*) were recorded continuously using a pneumotachometer with Hamilton Medical adult flow sensor (Hamilton Medical, Switzerland) connected to the ventilator circuit Y-piece. A laptop PC (Dell, Austin, TX, USA) was used with a National Instruments USB-6009 data acquisition unit and LabVIEW SignalExpress (National Instruments, Austin, TX, USA) to record the airway pressure and flow data with a sampling frequency of 100 Hz. Analysis was performed using MATLAB (R2014a, The Mathworks, Natick, MA, USA).

### Clinical protocol

Each patient included in the trial underwent a stepwise PEEP RM. Prior to the RM, patients were ventilated at a PEEP selected by the attending clinician, based on their experience. At this clinically selected PEEP, an arterial blood gas analysis was performed to determine PaO_2_. Before each RM, patients were sedated and paralyzed with muscle relaxants to prevent spontaneous breathing efforts. The specific protocol for the RM was

1. Decrease PEEP to 0 cmH_2_O (zero end-expiratory pressure, ZEEP)
2. Increase PEEP from 0 cmH_2_O in steps of 5 cmH_2_O until peak airway pressure (PIP) reached the limit of 45 cmH_2_O [30].
3. Maintain each PEEP level for 10–15 breathing cycles (~1 min)
4. From the maximum PEEP, reduce PEEP back to a clinically selected value in steps of 5 cmH_2_O.

The upper airway pressure was limited to 45 cmH_2_O to ensure patient safety. Further details of the clinical protocol can be found in the Additional file 1 (Section 1.0, Complete clinical protocol).

The 10–15 breathing cycles at each PEEP level provided a period of recruitment and stabilization based on the time frames observed in van Drunen et al. [31]. Patient-specific respiratory system elastance, *E*_*rs*_, for each PEEP level was calculated using the last breathing cycle before a PEEP increase. *E*_*rs*_ was identified using a single-compartment lung model [32]. This short time period at each PEEP level was designed to create a clinically feasible and efficient protocol that clinical staff would be more likely to follow. However, some patients may not be fully stabilized by this point.

### Data analysis

Respiratory system elastance, *E*_*rs*_, and resistance, *R*_*rs*_, were identified using the single-compartment lung model shown in Equation (1).

1 $$ {P}_{aw}={E}_{rs}\times V+{R}_{rs}\times Q+P0 $$

where *P*_*aw*_ is the airway pressure, *V* is lung volume, *Q* is flow rate and *P*0 is offset pressure. *E*_*rs*_ is identified from measured data using a multiple regression method [33]. Lung volume increase due to PEEP increase is known as end of expiratory lung volume (EELV) or dynamic functional residual capacity (dFRC) [34] and can be calculated from flow data as the amount of air not expired during PEEP increase.

Work of breathing (WOB) consists of two major components: work to overcome respiratory elastance and work to overcome airway resistance [35,36]. Thus, higher elastance requires more work to fill a given lung volume. Hence, minimum elastance is associated with reduced WOB [37-39]. The WOB at each PEEP is calculated using Equation (2) [37].

2 $$ \mathrm{W}\mathrm{O}\mathrm{B}=\left(\mathrm{average}\;\mathrm{inspiratory}\;\mathrm{pressure}-\mathrm{PEEP}\right)\times \mathrm{volume} $$

Relationships between *E*_*rs*_-*WOB* and *E*_*rs*_-dFRC were investigated using Pearson’s correlation coefficient.

### Identifying a clinically satisfactory PEEP

Initially, as PEEP is increased from ZEEP, *E*_*rs*_ falls as new lung volume is recruited faster than pressure builds up in the lung. At some point, little or no further recruitment occurs, and *E*_*rs*_ begins to rise with increasing PEEP, indicating that pressure above that PEEP level was unable to recruit significant new lung volume and is, instead, beginning to stretch already recruited lung [40]. Thus, while PEEP at minimum *E*_*rs*_ is optimal (*minimum*-*E*_*rs*_ PEEP), a slightly lower value may be more clinically palatable, as it reduces the chances of stretching the lung and causing injury.

A value of PEEP lower than *minimum*-*E*_*rs*_ PEEP, where *E*_*rs*_ is 5–10% above the observed minima, provides a margin of safety, while retaining the benefits of titrating PEEP to elastance. This point is labelled *inflection-E*_*rs*_ PEEP, as it represents a point of diminishing returns in the balance of recruitment safety. It is important to note that for any PEEP that exceeds minimum *E*_*rs*_, there will be a rise in *E*_*rs*_, risking overdistension. Thus, *inflection*-*E*_*rs*_ PEEP is proposed as a safety threshold.

The aim of this study is to test the feasibility of titrating PEEP to minimum *E*_*rs*_, by identifying the minimum during an RM. However, as *inflection-E*_*rs*_ PEEP provides similar benefits and may be safer and more acceptable, both points are presented in the results, for comparison.

## Results

**Figure 1:**
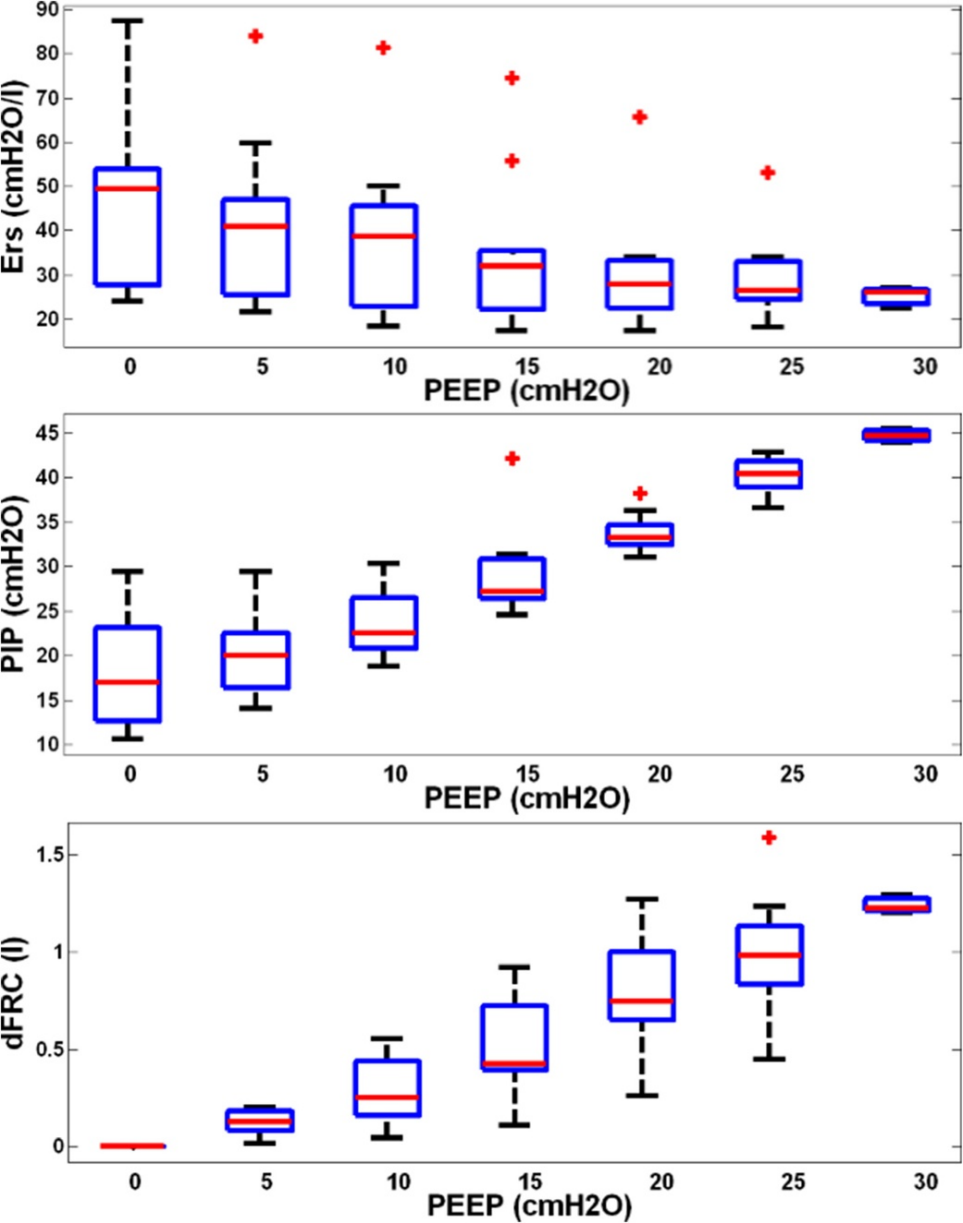
**Cohort respiratory data plotted against positive end-expiratory pressure (PEEP) level.** The top panel shows the distribution of patient-specific elastance (*E*_*rs*_) across the 10 patients at each PEEP level. The middle panel shows peak inspiratory pressure (PIP) and the bottom plot dynamic functional residual capacity (dFRC). Red cross outliers: the outliers in *E*_*rs*_ are mainly from patient 5. PEEP levels were classified by rounding to the nearest 5 cmH_2_O.

Demographics and clinical details of patients are shown in Table 1. Figure 1 shows the distribution of respiratory elastance (*E*_*rs*_) (top panel), peak inspiratory pressure (PIP) (middle panel) and dynamic functional residual capacity (dFRC) (bottom panel) across the cohort for each PEEP. Interestingly, paired PaO_2_ measurements, before and 30 min after RM, indicated a statistically non-significant reduction in arterial oxygen by a median 11 mmHg [interquartile range (IQR) 0–15] per patient, following the RM (*p* = 0.21, Wilcoxon signed-rank test). Cohort median PaO_2_ values for the cohort were 84.0 mmHg [IQR 73.0–114.0] and 77.5 mmHg [IQR 68.0–86.0] for before and after RM, respectively.

**Table 1:** **Patient demographics and clinical details**

Patient	Sex	Age	Clinical diagnostic	PF ratio (mmHg)	APACHE II	SAPS II	FiO_2_	Clinically selected PEEP	Heart rate (bpm)^a^	Blood pressure systolic (mmHg)^a^	Blood pressure diastolic (mmHg)^a^	PaO_2_ before RM (mmHg)	PaO_2_ 30 min after RM (mmHg)
1	F	61	Peritonitis, COPD	209	18	41	0.35	10	73	122	64	73	60
2	M	22	Trauma	170	12	25	0.50	12	93	143	72	85	73
3	M	55	Aspiration	223	21	44	0.35	10	87	131	81	78	76
4	M	88	Pneumonia, COPD	165	24	42	0.40	10	98	168	51	66	56
5	M	59	Pneumonia, COPD	285	23	50	0.40	12	91	102	63	114	79
6	M	69	Trauma	280	18	44	0.35	11	89	118	51	98	118
7	M	56	Legionnaires	265	18	34	0.55	7.5	102	165	70	146	68
8	F	54	Aspiration	303	23	49	0.40	12	104	172	71	121	106
9	M	37	H1N1, COPD	183	13	21	0.40	10	96	125	55	73	86
10	M	56	Legionnaires, COPD	237	18	33	0.35	10	64	112	50	83	83
Median [IQR]				230 [183–280]								84.0 [73.0–114.0]	77.5 [68.0–86.0]

^a^Values are in median.

*Abbreviations*: *APACHE II* acute physiology and chronic health evaluation II, *bpm* beats per minute, *COPD* chronic obstructive pulmonary disease, *FiO*_*2*_ fraction of inspired oxygen, *IQR* interquartile range, *PaO*_*2*_ partial pressure of oxygen in arterial blood, *PEEP p*ositive end-expiratory pressure, *PF ratio* partial pressure of oxygen in arterial blood/fraction of inspired oxygen, *RM* recruitment manoeuvre, *SAPS II* simplified acute physiology score II.

**Figure 2:**
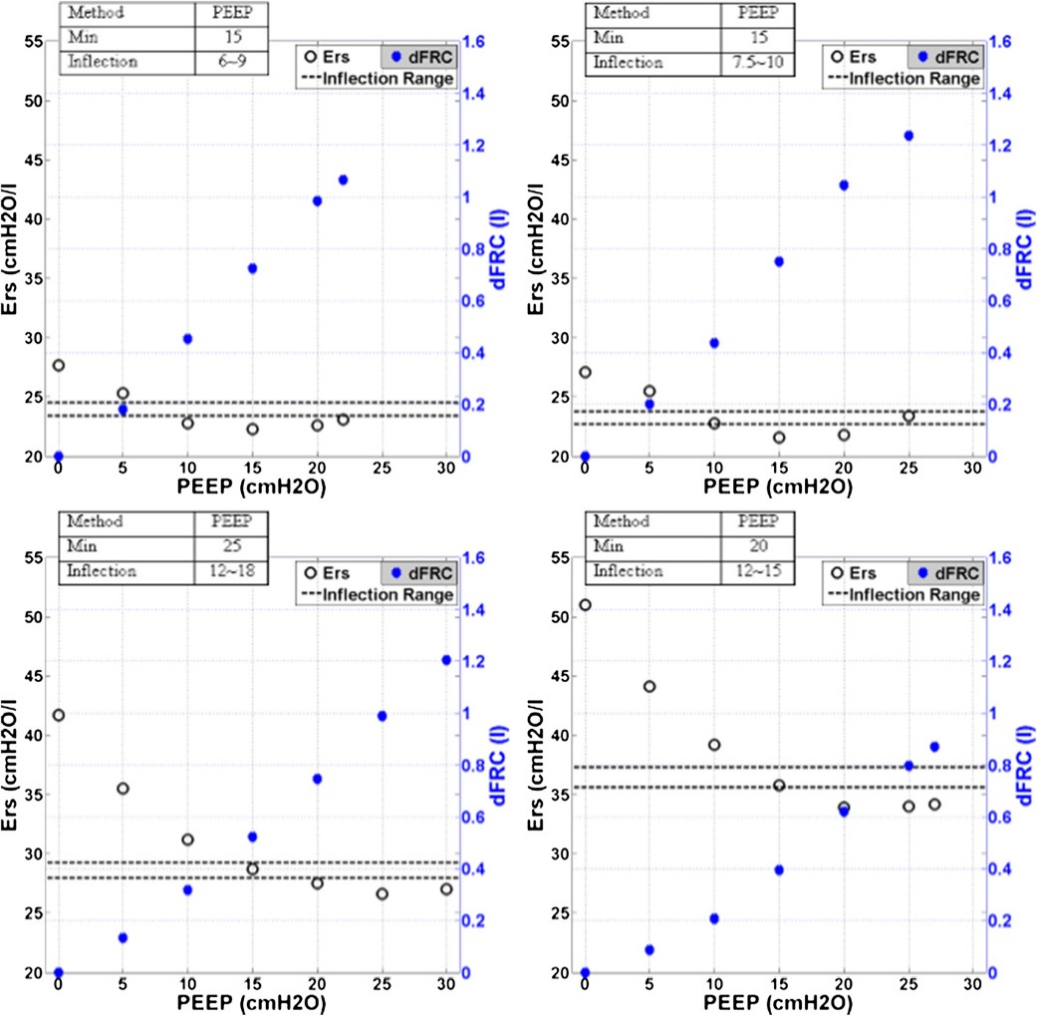
**Respiratory mechanics as a function of positive end-expiratory pressure (PEEP).** Top left panel, patient 2; top right panel, patient 6; bottom left panel, patient 8; bottom right panel, patient 10. PEEP derived from *minimum-E*_*rs*_ and *inflection-E*_*rs*_ method are as indicated. The dashed line is the range for *inflection-E*_*rs*_. The dynamic functional residual capacity (dFRC) is also indicated.

**Figure 3:**
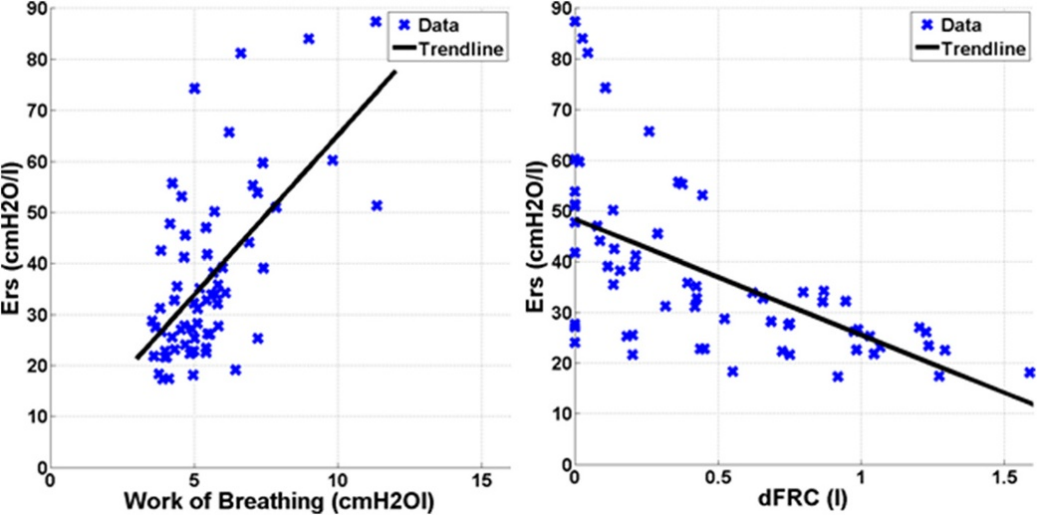
**Pearson’s correlation.** (Left) Elastance-work of breathing (*E*_*rs*_-*WOB*), *R* = 0.62. (Right) Elastance-dynamic functional residual capacity (*E*_*rs*_-*dFRC*), *R* = −0.62.

Figure 2 shows patient-specific respiratory system elastance (*E*_*rs*_) and dFRC with increasing PEEP for Patients 2, 6, 8 and 10. The dashed lines shown in Figure 2 are the range where *inflection-E*_*rs*_ is located (5–10%, above minimum *E*_*rs*_). The optimal PEEP using *minimum-E*_*rs*_ and *inflection-E*_*rs*_ are also indicated. The patient-specific *E*_*rs*_ and dFRC with increasing PEEP for all 10 patients are included in the Additional file 1 (Section 2.0, Additional results) provided with the manuscript. Figure 3 presents calculated WOB (left) and dFRC (right) data against *E*_*rs*_. The Pearson correlation of coefficient between *E*_*rs*_ and WOB is *R* = 0.62, and *R* = −0.62 for dFRC.

**Figure 4:**
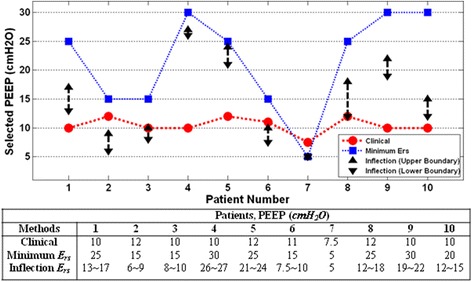
**Positive end-expiratory pressure (PEEP) selection comparison.** Comparison between clinical selection, *minimum-E*_*rs*_ and *inflection-E*_*rs*_.

Figure 4 compares clinically selected PEEP by attending clinicians during MV therapy with *minimum-E*_*rs*_ and *inflection-E*_*rs*_ values. Across the cohort, these clinical values were within a much narrower range than those selected using patient-specific elastance. Complete patient-specific respiratory mechanics data at each PEEP can be found in the Additional file 1 (Section 2.0, Additional results).

## Discussion

### Influence of PEEP in patient’s *E*_*rs*_, PIP and dFRC

Wide ranges of *E*_*rs*_, PIP and dFRC were observed across all patients and PEEPs. These results reflect the heterogeneity of ARDS patient condition and response to PEEP. It is clear from these results that standardizing PEEP selection is difficult [15], and there is a need for patient-specific approach. Examining the *E*_*rs*_ and dFRC across PEEP levels, it was found that all 10 patients included in this study had a different range of *E*_*rs*_, dFRC as well as a patient-specific *minimum-E*_*rs*_ PEEP within the tested range (see Additional file 1 (Section 2.0, Additional Results)).

At ZEEP, *E*_*rs*_ is relatively high and variable across patients (Figure 1). As PEEP is increased, *E*_*rs*_ reduces at a rate that is patient-specific (Figure 2). This trend of *E*_*rs*_ reduction with PEEP increase matches previous PEEP titration studies in animal subjects [25-27]. This *E*_*rs*_ trend with PEEP suggests recruitment, with increase of lung volume outweighing increase in pressure. The dFRC shown in Figure 1 (bottom panel) continued to increase with PEEP, following a sigmoidal curve. This sigmoidal curve indicates overstretching of the lung that occurs at higher PEEP.

Increasing PEEP past the *minimum-E*_*rs*_ point risks overstretching of healthy lung units even at lower PEEP and airway pressures [19]. In Figure 2, patients 2 and 6 (top panels) show examples of *E*_*rs*_ increasing after the minimum, indicating potential stretching of lung units. However, the minimum is not always obvious or present, as was the case for patients 8 and 10, shown in the bottom panels of Figure 2. In this case, *E*_*rs*_-PEEP curve became very flat without further significant reductions in *E*_*rs*_ with increases of PEEP from 15 to 30 cmH_2_O. Thus, identifying a point of diminishing returns (*inflection-E*_*rs*_) is potentially a safer approach to PEEP selection.

A moderate correlation between *E*_*rs*_ and both WOB and dFRC was observed. This result was expected as elastance relates changes in volume to changes in pressure, and WOB is the product of these two quantities across a breath. Thus, higher elastance requires more work to fill a given lung volume. This result suggests an additional potential benefit to selecting PEEP for minimum *E*_*rs*_ to WOB for patients can be minimized if ventilated at the point of minimum elastance.

WOB is normally assessed when the patient is spontaneously breathing and may seem pointless in this study where patients were paralyzed. However, these patients slowly resume spontaneous breathing effort as muscle relaxant wears off. Thus, by setting PEEP to minimize a component of WOB could potentially benefit these patients as they regain spontaneous breathing ability.

### General observations

In this study, it was found that PEEP selected using *minimum* or *inflection*-*E*_*rs*_ were different from clinically selected PEEP. *Inflection-E*_*rs*_ selected PEEP was both above and below clinically selected values. This results show a clear variation between these methods. In particular, the clinically selected PEEP was lower than either *minimum*-*E*_*rs*_ or *inflection-E*_*rs*_ PEEP in patients with chronic obstructive pulmonary disease (COPD). This result implies that patients with COPD potentially benefit from high PEEP ventilation. It was also found that at higher PEEP of 15–20 cmH_2_O, the overall maximum PIP remains around 45 cmH_2_O, indicating patients were ventilated at a safe level [30]. However, selecting PEEP is a trade-off in minimizing lung pressure and potential damage versus maximizing recruitment.

**Figure 5:**
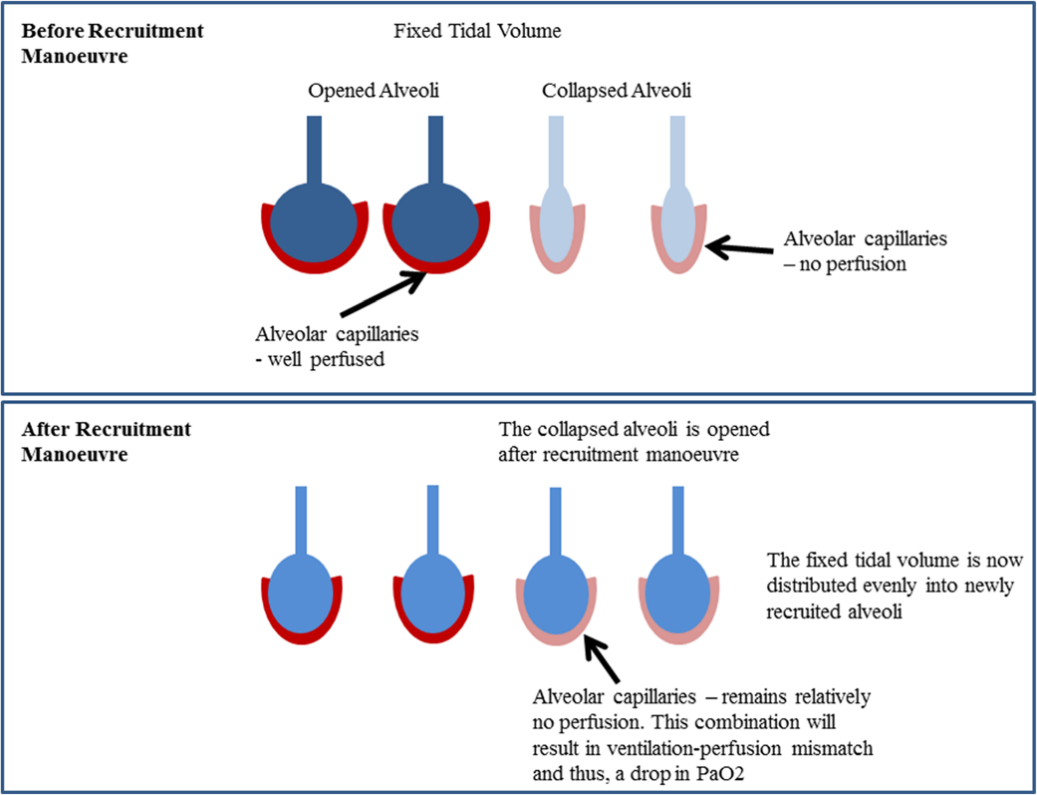
**Ventilation with no perfusion.** This condition is due to insufficient blood flow into the ‘newly opened’ alveoli capillaries. A darker colour shows better perfusion (red) and/or better distribution (blue) of air.

A total of 7 of 10 patients’ PaO_2_ decreased after the RM by 11 mmHg [IQR 0–15] (*p* = 0.21, Wilcoxon signed-rank test). While statistically non-significant, this result is worth considering as it is both counterintuitive and contradictory to some studies where significant increases in PaO_2_ after RM were observed (*p* < 0.05) [30,41]. Studies by Tusman et al. [41] and Gattinoni et al. [30] have shown improvement in oxygenation (up to 20%) due to alveolar recruitment. However, other studies have shown no significant differences or reductions in PaO_2_ of up to 10% [42-44]. To explain this observed reduction in our trial, we hypothesize that between RM and arterial blood gas analysis, there was insufficient lung perfusion as shown in Figure 5.

Prior to the RM, a fixed tidal volume was distributed between recruited lung units. However, after RM, the same tidal volume would be distributed amongst a larger number of lung units. The newly recruited alveoli are potentially poorly perfused with blood, resulting in a transient lower oxygen transfer to blood and consequently a reduction of observed PaO_2_. In addition, the study by Oczenski et al. [42] also showed that maintaining post recruitment manoeuvre PEEP similar to baseline PEEP may not benefit long-term oxygenation improvement, and there is a need to titrate to ‘optimal’ PEEP.

### Titrating PEEP

The goal of this study is to investigate the feasibility of using an RM to identify patient-specific lung elastance that can be used to titrate a more optimal PEEP. Elastance, *E*_*rs*_, was calculated during the increase-PEEP section of the RM. Specifically, data from a single inspiration were used, taken from a breath approximately 10 ~ 15 breathing cycles following PEEP increase, to allow stabilization. This procedure is different to the animal studies by Carvalho et al. [25], Suarez-Sipmann et al. [26] and Lambermont et al. [27]. In these studies, elastance (or compliance) was calculated during a PEEP titration manoeuvre after a significant period of time for recruitment [45].

In general, this study has shown that titrating an ‘optimal’ PEEP for each patient, based on respiratory mechanics, is feasible. Optimal, in this case, is defined as minimum (or near-minimum) elastance, as this point enables maximum recruitment with minimum risk of lung damage. The proposed *inflection-E*_*rs*_ is essentially an alternate means of finding a potentially much lower PEEP value that further reduces any unintended potential damage with minimal loss from the optimal minimum *E*_*rs*_. However, the clinical benefit of a patient ventilated at PEEP selected using both *inflection-E*_*rs*_ and *minimum-E*_*rs*_ was not evaluated in this study and warrants further investigation.

### Study limitations and recommendations

#### Protocol in hemodynamic management

During the RM, there were no standardized protocols for managing hemodynamic stability. Thus, some patients were given hemodynamic therapies such as fluid resuscitation and vasopressors that may have impacted upon the results of this study. However, the responses to these therapies were patient-specific, and the patient numbers were too low in this proof-of-concept study to correlate specific elastance responses to the use of these therapies. Equally, it is important to note that the study was primarily focused on proving the metric, capturing the differences across PEEP levels for a range of patients and conditions, which has been shown. For information, Additional file 1 (Section 3.0, Hemodynamic stability) contains the patient-specific details on hemodynamic therapies that were not part of the study protocol.

#### Muscle paralysis

Spontaneous breathing efforts can affect the identified respiratory elastance, *E*_*rs*_, as the muscular effort can increase or decrease the apparent mechanical properties. Thus, a muscle relaxant was used in this trial to ensure patients were suitably paralyzed, and *E*_*rs*_ could be identified reliably during fully controlled ventilation. However, the use of muscle relaxants for this purpose may be clinically undesirable and could be seen as a limitation of this method. This concern was also raised in the RCT by Pintado et al. [28], in which the authors suggested that finding the maximum compliance PEEP (minimum *E*_*rs*_-PEEP) can be difficult in patients and required muscle relaxants [28]. This potential limitation could be overcome by using oesophageal pressure measurement to remove the spontaneous breathing signal [46].

#### Monitoring *E*_*rs*_ and recruitment manoeuvre design

For *E*_*rs*_ monitoring, Lambermont et al. [27] allowed 15 min for stabilization time, Suarez-Sipmann et al. [26] allowed 10 min and Carvalho et al. [25] only used 3 min. The clinical study conducted by Suter et al. [23] allowed 15–20 min for stabilization during increasing PEEP whereas Pintado et al. [28] did not specify the stabilization time period, with the PEEP titration performed during increasing PEEP. Thus, the *E*_*rs*_ response observed in this study may be limited to PEEP-induced recruitment and not time-dependent recruitment (if stabilization was not achieved). This limitation is also outlined in the animal trials by van Drunen et al. [47]. The 10–15 breathing cycles stabilization period allowed for this trial may not be sufficient for all patients as alveoli recruitment is both PEEP- and time-dependent. Thus, *E*_*rs*_ and the suggested PEEP from this protocol may be slightly higher than other studies as there is no prior recruitment. The progression of *E*_*rs*_ with time and PEEP should be investigated in parallel with a longer stabilization time. An ‘optimal’ stabilization time period could be established because a shorter stabilization period will significantly reduce the protocol burden, while a longer stabilization may achieve more ‘complete’ recruitment.

A further potential limitation is that several studies have reported that not all patients benefit from RM. In particular, Fan et al. [48], Pelosi et al. [49] and Guerin et al. [44] have reported conflicting results, where the benefit of a RM is dependent of the patient-specific disease state, as well as the design of the RM. Thus, assessing the efficacy of specific forms of RM design should be considered in future clinical trials. In particular, a recruitment manoeuvre of a specific design and its corresponding effect towards cardiovascular, respiratory system and hemodynamic response should be monitored closely with longer period at additional time steps (5, 15, 30, 45, 60, 90 and 120 min after the RM). Equally, the specific design (for example, maximum allowable PEEP or PIP) of RM needs to be adjusted based on the severity of ARDS.

## Conclusions

This study has shown that titrating an ‘optimal’ PEEP for each patient, based on respiratory mechanics, is feasible. A patient-specific approach is desirable as respiratory mechanics response to PEEP was shown to be quite variable between patients. *Minimum*-*E*_*rs*_ or *inflection*-*E*_*rs*_ PEEP can be found for every patient and thus provide an opportunity to individualize PEEP settings. The patient-specific respiratory elastance, *E*_*rs*_, was shown to be correlated with work of breathing and dynamics functional residual capacity, further indicating its clinical relevance and potential. The approach presented in this proof-of-concept study offers potential to improve clinical insight and guidance in selecting PEEP.

## Additional file

Additional file 1: **A supplementary document that consist of several information.** The information are (1) complete clinical protocol, (2) additional results, (3) hemodynamic stability and (4) independent patient study—patient 9 (H1N1).

## Abbreviations

APACHE: Acute physiology and chronic health evaluation
ARDS: Acute respiratory distress syndrome
COPD: Chronic obstructive pulmonary disease
dFRC: Dynamic functional residual capacity
*E*_*rs*_: Patient-specific respiratory elastance
FiO_2_: Fraction of inspired oxygen
ICU: Intensive care unit
IQR: Interquartile range
MV: Mechanical ventilation
*P*0: Offset pressure
PaO_2_: Partial pressure of oxygen in arterial blood
*P*_*aw*_: Airway pressure
PEEP: Positive end-expiratory pressure
PF ratio: PaO_2_/FiO_2_
*Q*: Flow
RM: Recruitment manoeuvre
*R*_*rs*_: Resistance
SAPS: Simplified acute physiology score
SIMV: Synchronized intermittent mandatory ventilation
*V*: Volume
VILI: Ventilator-induced lung injury
*V*_*t*_: Tidal volume
WOB: Work of breathing
ZEEP: Zero PEEP
