# Supplementary material for: Feasibility of titrating PEEP to minimum elastance for mechanically ventilated patients
Source: Pilot Feasibility Stud. 2015 Mar 21;1:9. doi: 10.1186/s40814-015-0006-2 (PMC5395899; doi:10.1186/s40814-015-0006-2)
Supplement: Additional file 1: — A supplementary document that consist of several information. The information are (1) complete clinical protocol, (2) additional results, (3) hemodynamic stability and (4) independent patient study—patient 9 (H1N1). [file 40814_2015_6_MOESM1_ESM.doc]

**Additional File 1**

**1.0 Complete Clinical Protocol**

The following presents the study protocol:

**Inclusion Criteria:**

1. Patient on mechanical ventilation.
2. Patients diagnosed with ARDS by attending clinicians (Acute onset, findings of bilateral infiltrates on chest radiograph, absence of left side heart failure and *PaO_2_/FiO_2_* (PF ratio) between 150-300 *mmHg*).

**Exclusion criteria:**

1. Patients who were likely to be discontinued from MV in 24 hours.
2. Patients with age < 16.
3. Moribund and/ or not expected to survive for greater than 72 hours.
4. Patient who are minimally sedated. These patients have spontaneous breathing effort and additional sedation may prolong the length of MV

Written informed consent was obtained from the patient/ family members or relatives for the trials and publication of the clinical details.

The trials and use of the data were approved by the New Zealand, South Island Regional Ethics Committee. The trial is registered with Australian New Zealand Clinical Trials Registry (ACTRN 12611001179921).

**Mechanical Ventilator and Settings:**

All patients were ventilated using Puritan Bennett PB840 ventilator (Covidien, Boulder, CO, USA) with volume control (tidal volume, *V_t_* = 6~8 ml/kg, synchronized intermittent mandatory ventilation (SIMV) mode.

**Clinical Protocol:**

1. Eligible patient will undergo a step-wise PEEP increase recruitment manoeuvre (RM).
2. Before the start RM, the pneumotachometer and data acquisition system is attached to the patient-ventilator Y-piece.

The pneumotachometer records the patient pressure and flow profile throughout the clinical protocol. These pressure and flow data are used to estimate patient elastance (*E­_rs_*) at different PEEP.

1. Before RM, patients were sedated and paralyzed with muscle relaxants to prevent spontaneous breathing efforts.

Patients who are fully sedated will not have spontaneous breathing effort and thus respiratory mechanics can be easily estimated using airway pressure and flow measurement .

1. The arterial blood gas (ABG) was recorded at clinically selected PEEP.

The ‘clinically selected PEEP’ is the PEEP selected by the attending doctor as they see fit prior to the RM.

1. The clinically selected PEEP is first reduced to 0 *cmH_2_O* (Zero end-expiratory pressure, ZEEP)
2. During RM, PEEP was increased stepwise of 5 *cmH_2_O* from ZEEP.
3. PEEP is increased until peak airway pressure (PIP) reaches a limit of 45 *cmH_2_O*

PIP of 45*cmH_2_O* is used a threshold to ensure no overdistension as shown in study by [^3^](#_ENREF_3).

1. Each PEEP was maintained for 10~15 breathing cycle before increasing to the higher PEEP.

PEEP induced alveoli recruitment is known to be time-dependant. The 10~15 breaths period allows recruitment to fully occur and allows the lung to achieve viscoelastic stabilisation.

1. After reaching maximum allowable PEEP at which maximum PIP is limited to 45*cmH_2_O*, PEEP was reduced stepwise to clinically selected PEEP.
2. 30 minutes after RM, the patient’s arterial blood gas was sampled.

This ABG measurement is to compare ABG before recruitment to examined the effect of recruitment manoeuvre towards oxygenation patients.

Figure E1 shows the PEEP step increment during RM.


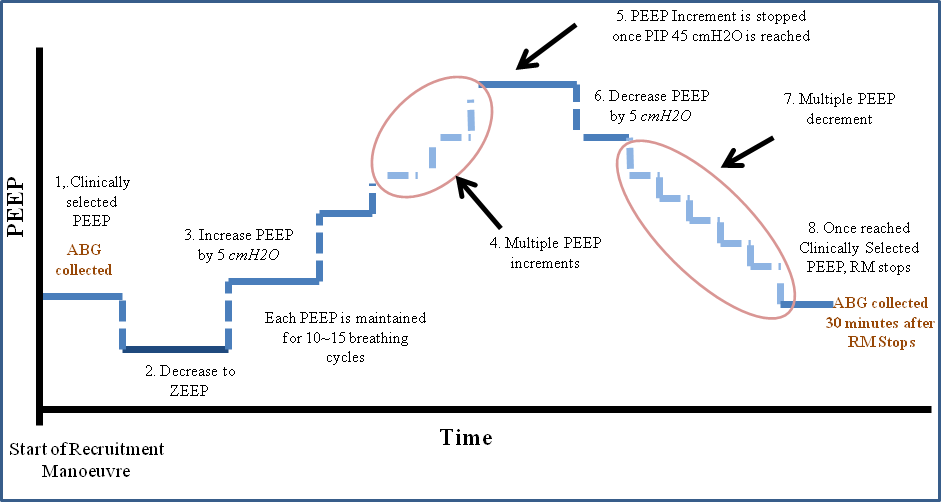


**Figure E1:** PEEP increment during recruitment manoeuvre. Respiratory Elastance is identified during PEEP increment.

**2.0 Additional Results:**

Table E1 below shows patient-specific Respiratory elastance (*E_rs_*), dynamic functional residual capacity, work of breathing (WOB) and Elastance Pressure (P*ev* = *E_rs_* × *V*) at each PEEP are shown below. Pev is a surrogate of plateau pressure during end-of inspiratory pause [^4^](#_ENREF_4), and can be used as an indicator for overdistension. As noted in previous studies, setting PEEP with plateau pressure of less than 30~35 *cmH_2_O* is able to avoid barotrauma.

**Table E1:** Patient-specific *E_rs_*-dFRC-WOB-Pev vs PEEP

| **Patient 1** | |
| --- | --- |
| 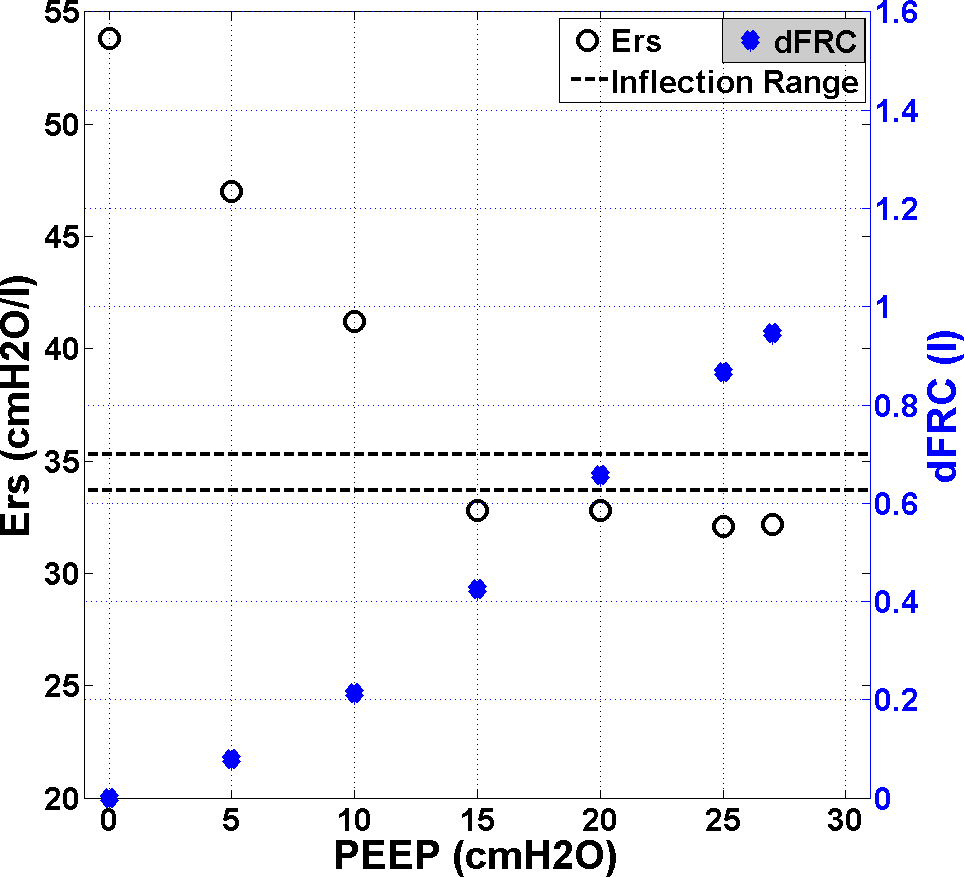 | 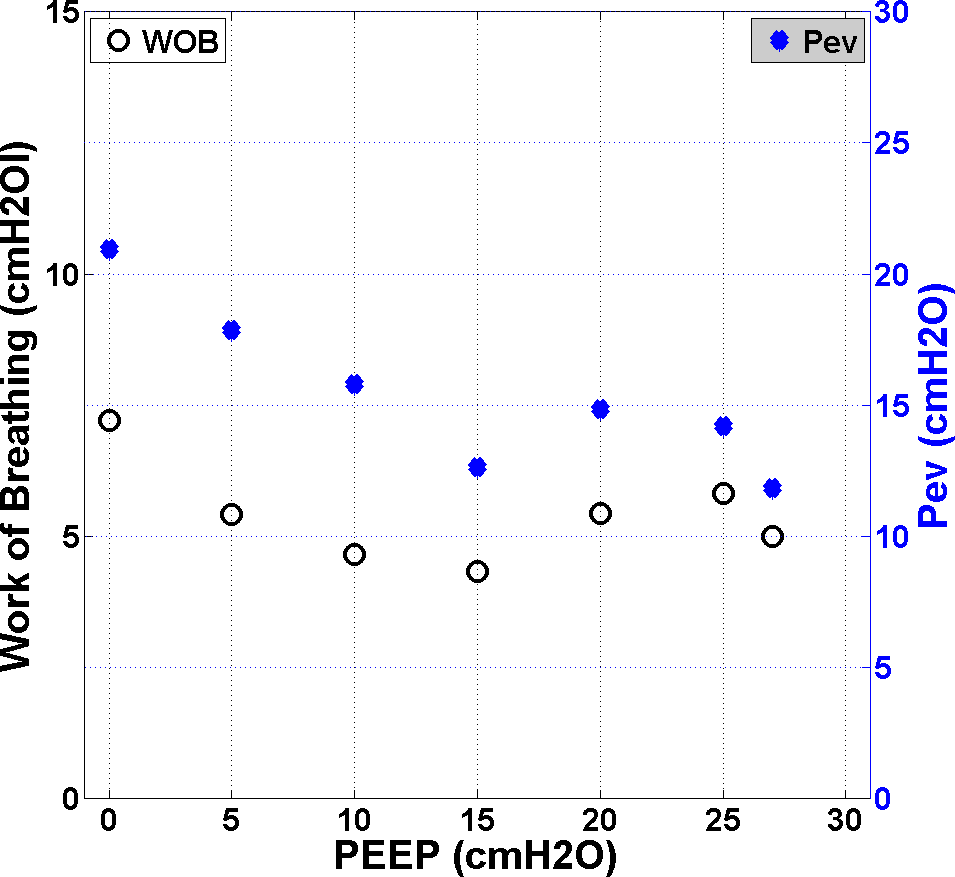 |
| **Patient 2** | |
| 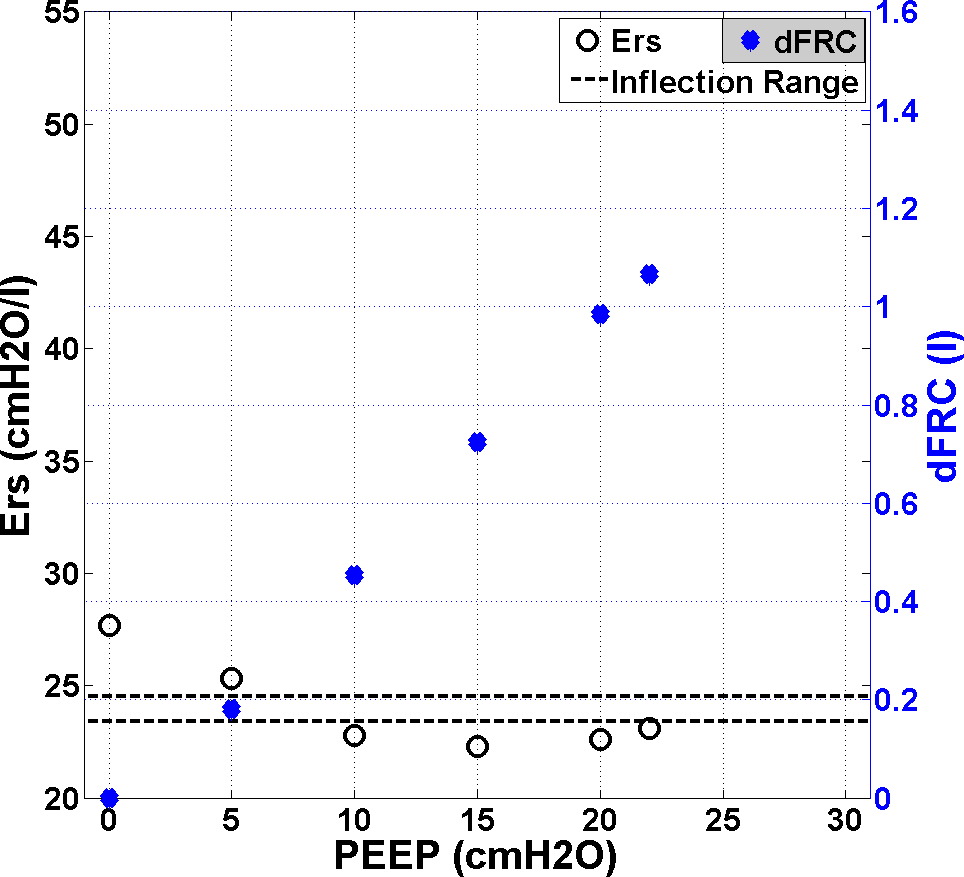 | 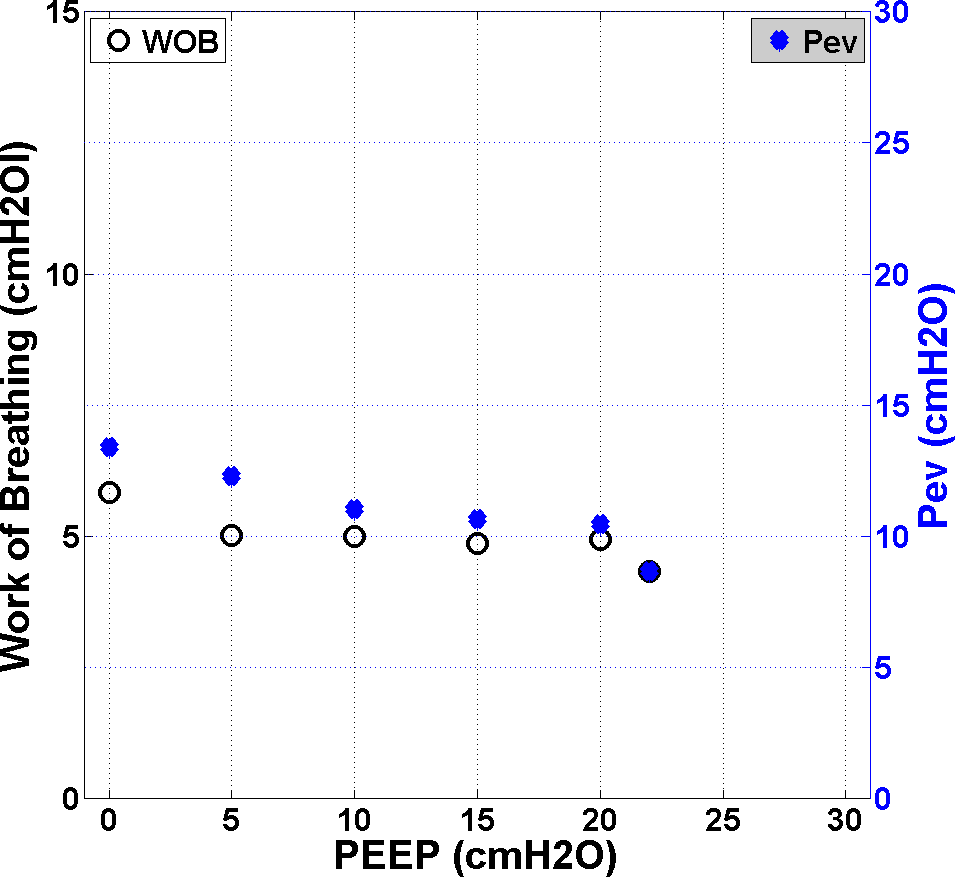 |
| **Patient 3** | |
| 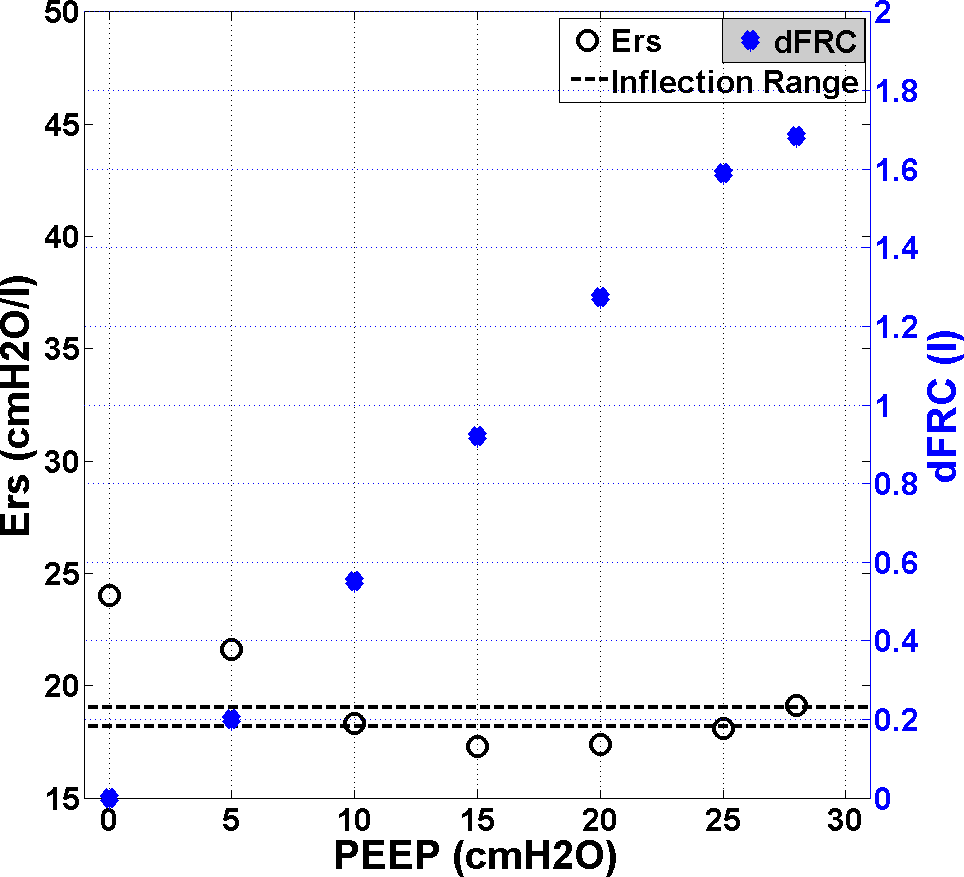 | 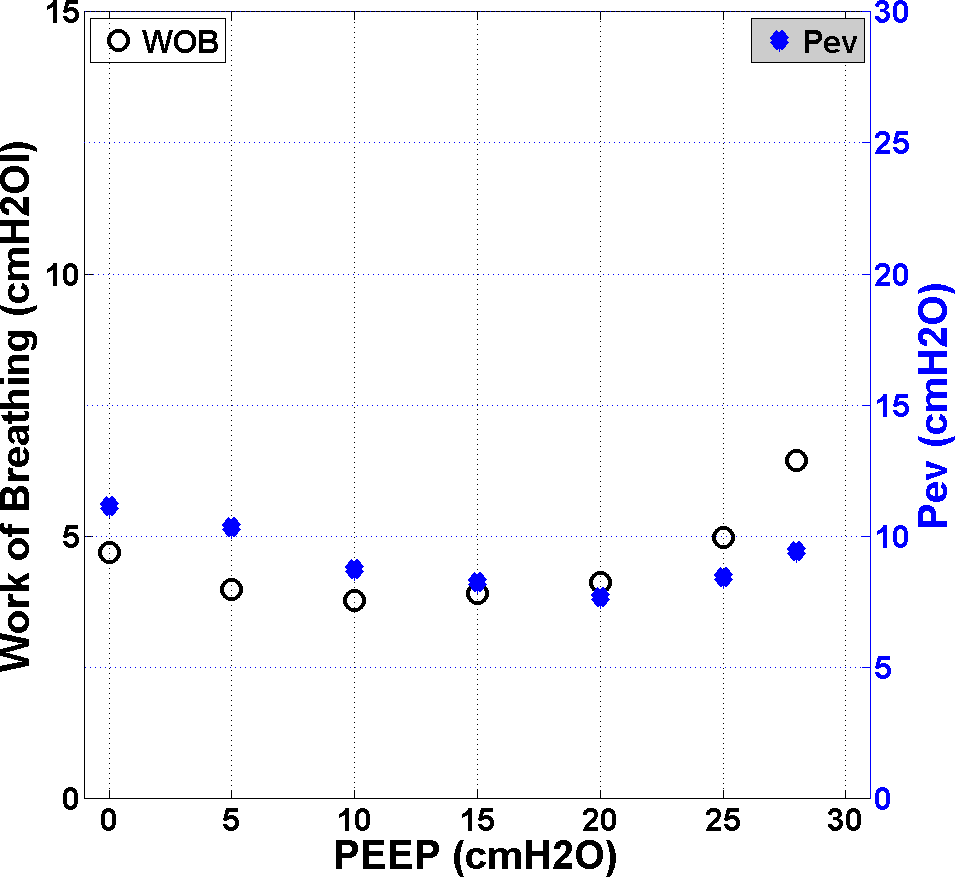 |
| **Patient 4** | |
| 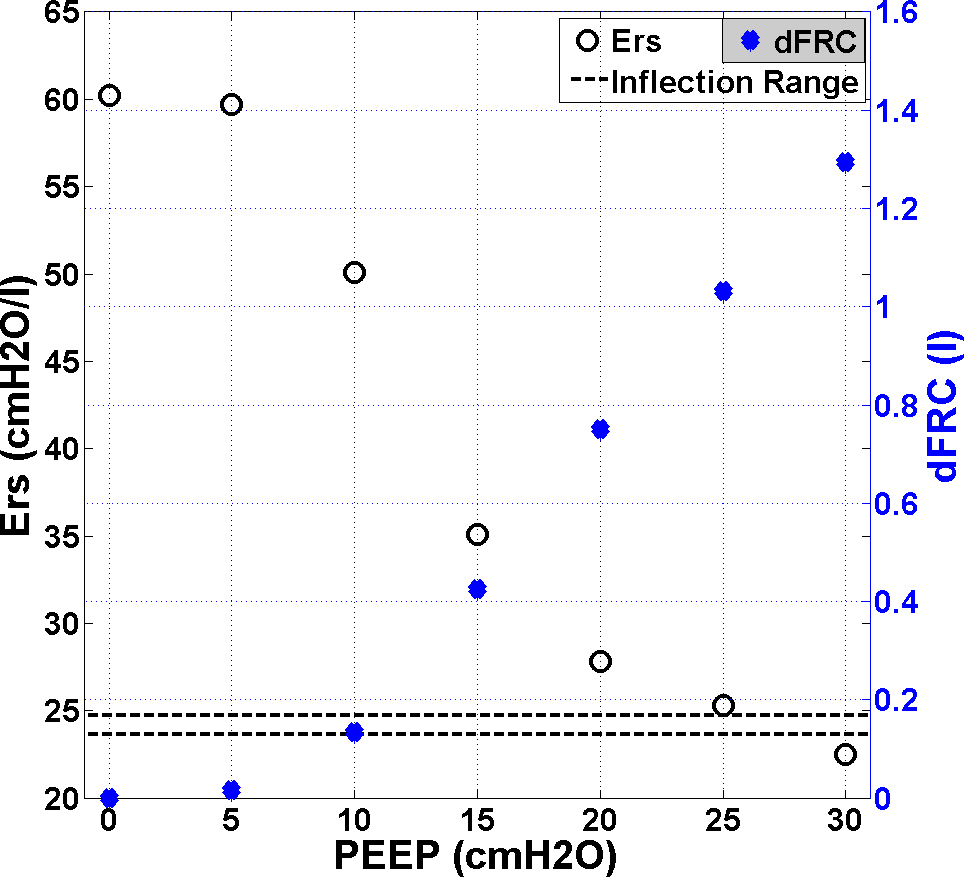 | 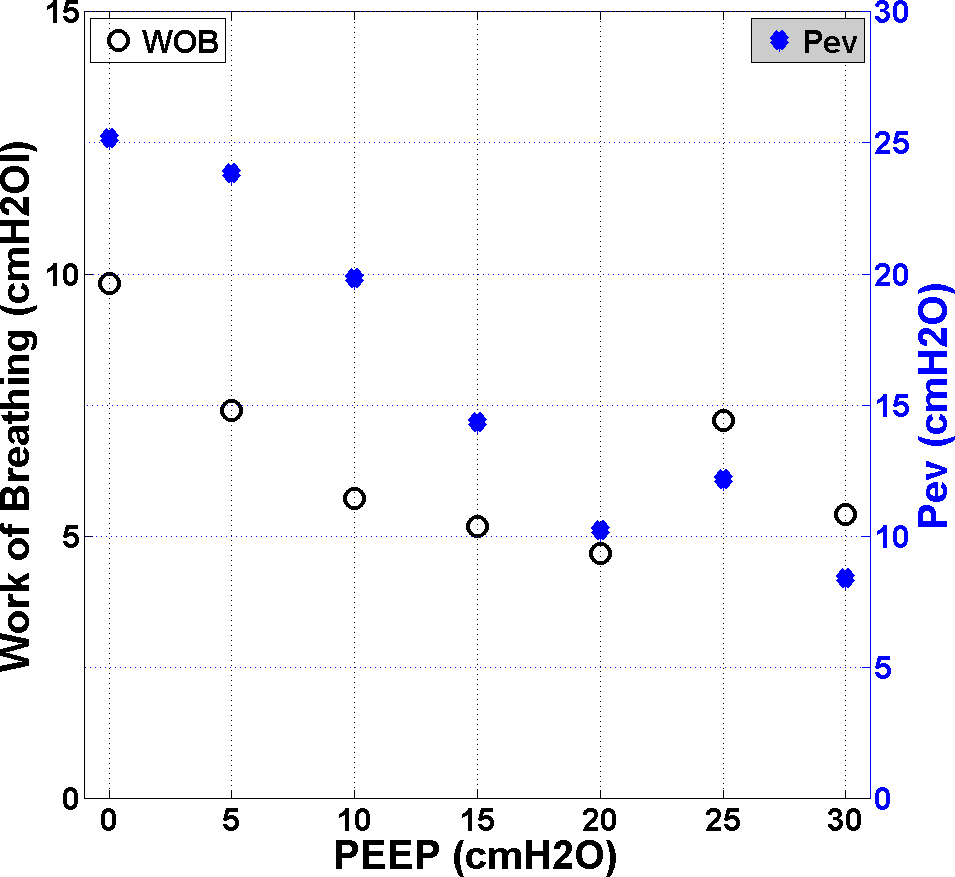 |
| **Patient 5** | |
| 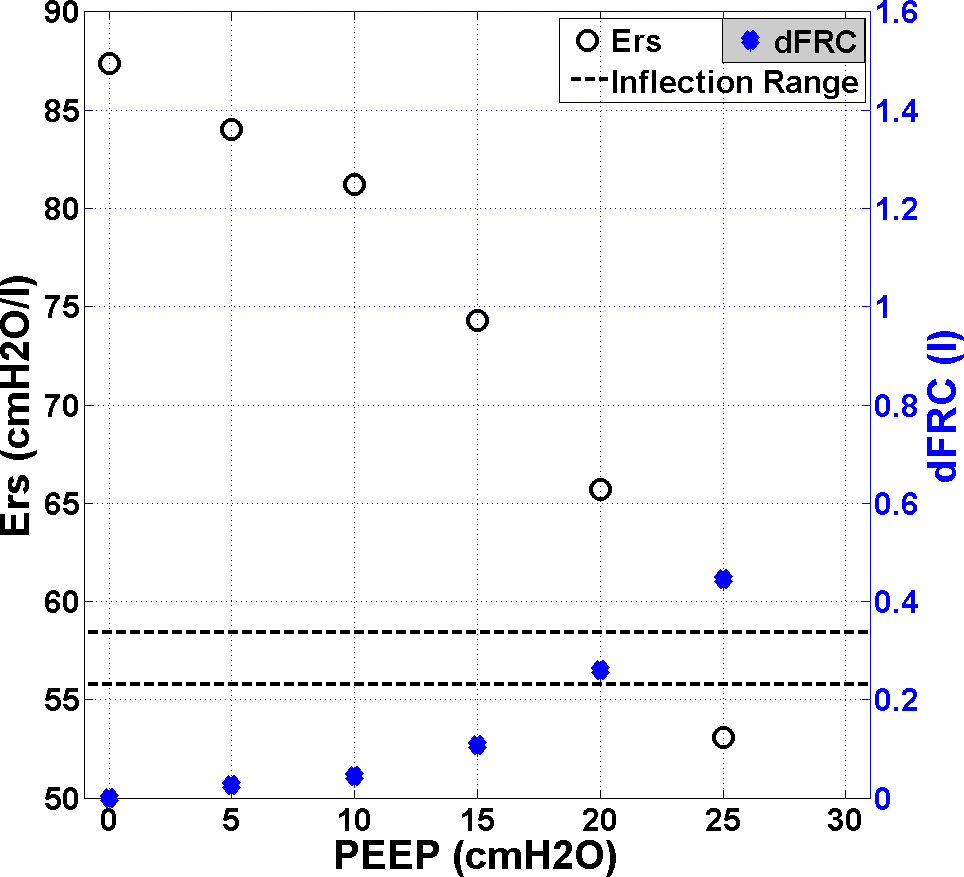 | 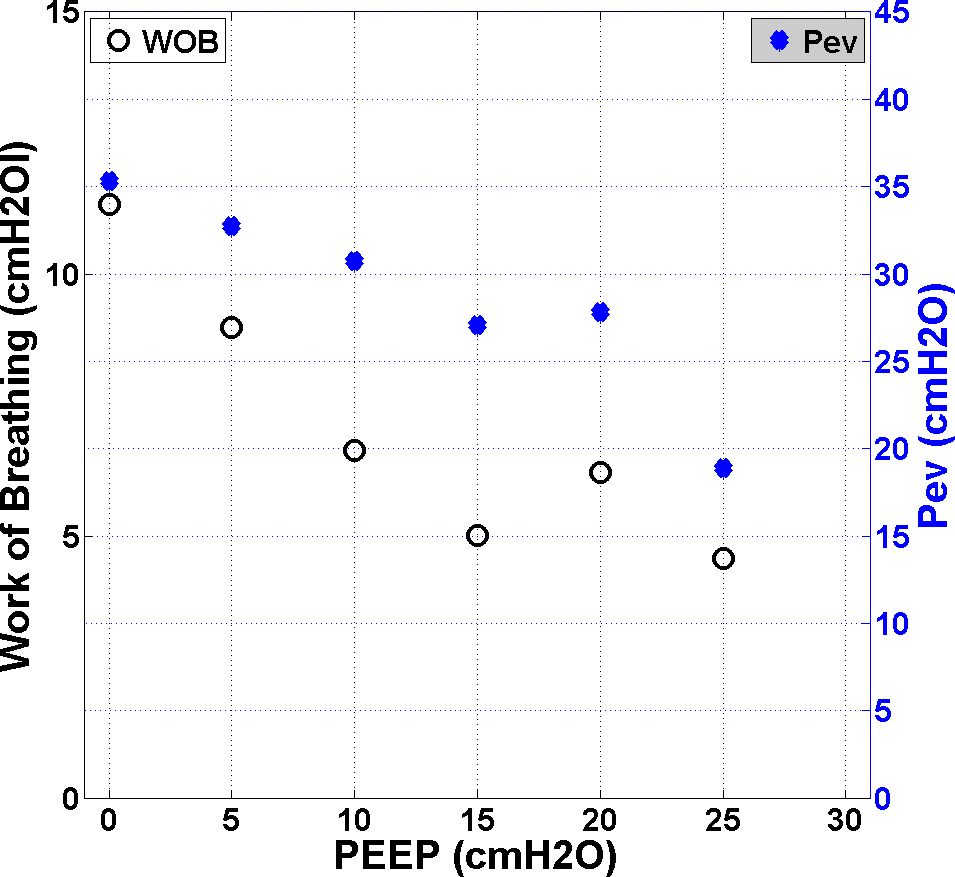 |
| **Patient 6** | |
| 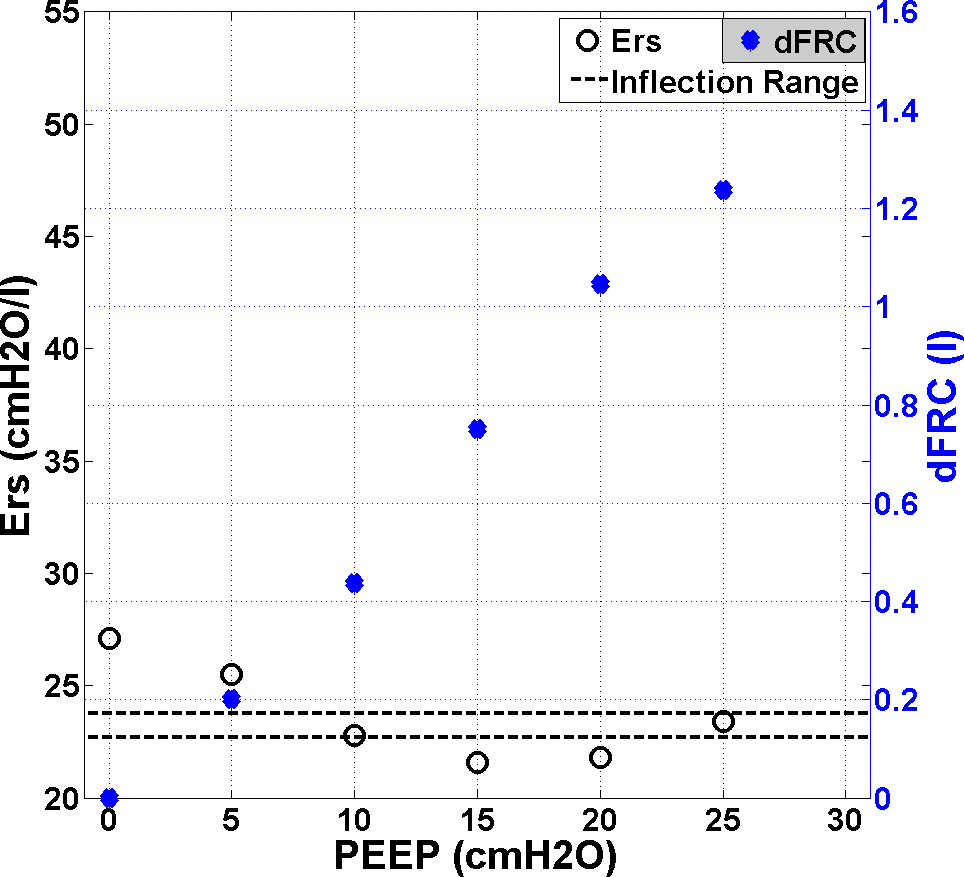 | 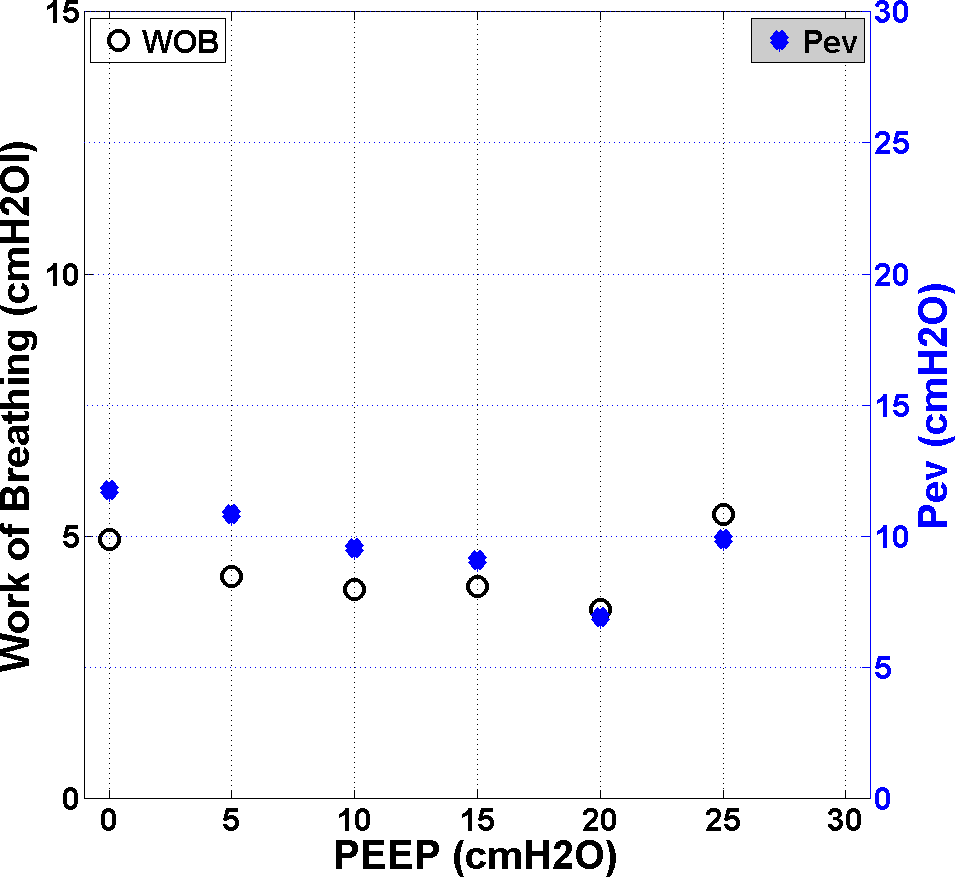 |
| **Patient 7** | |
| 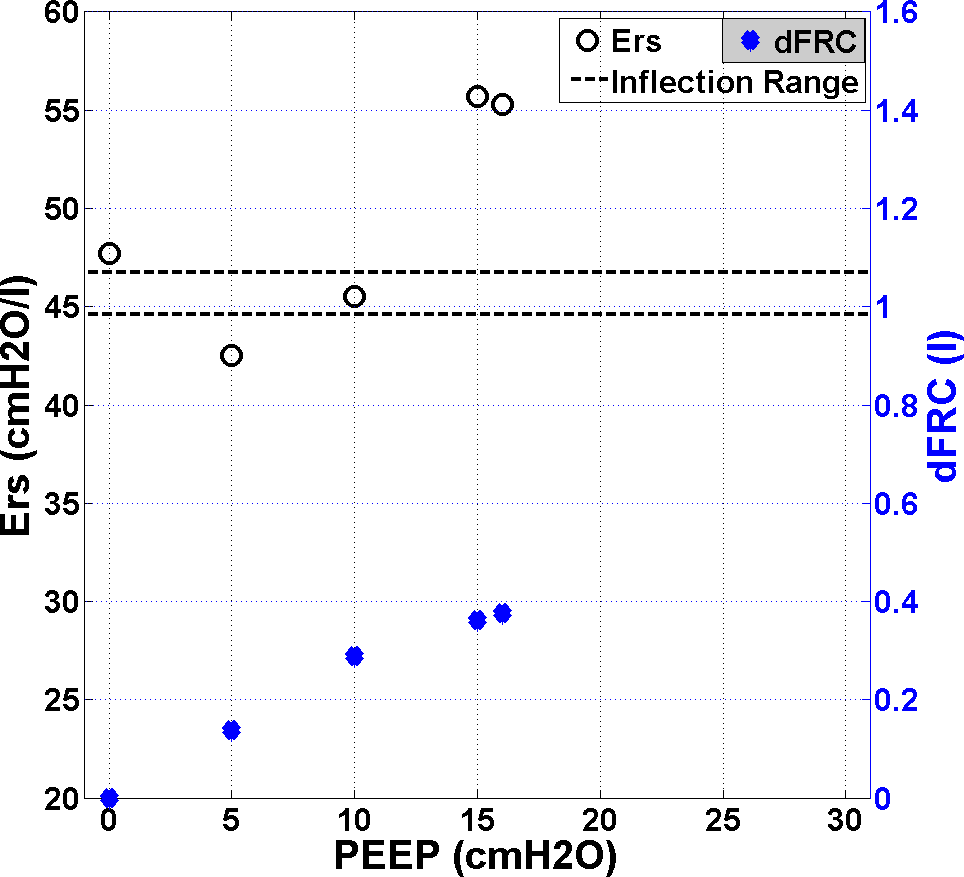 | 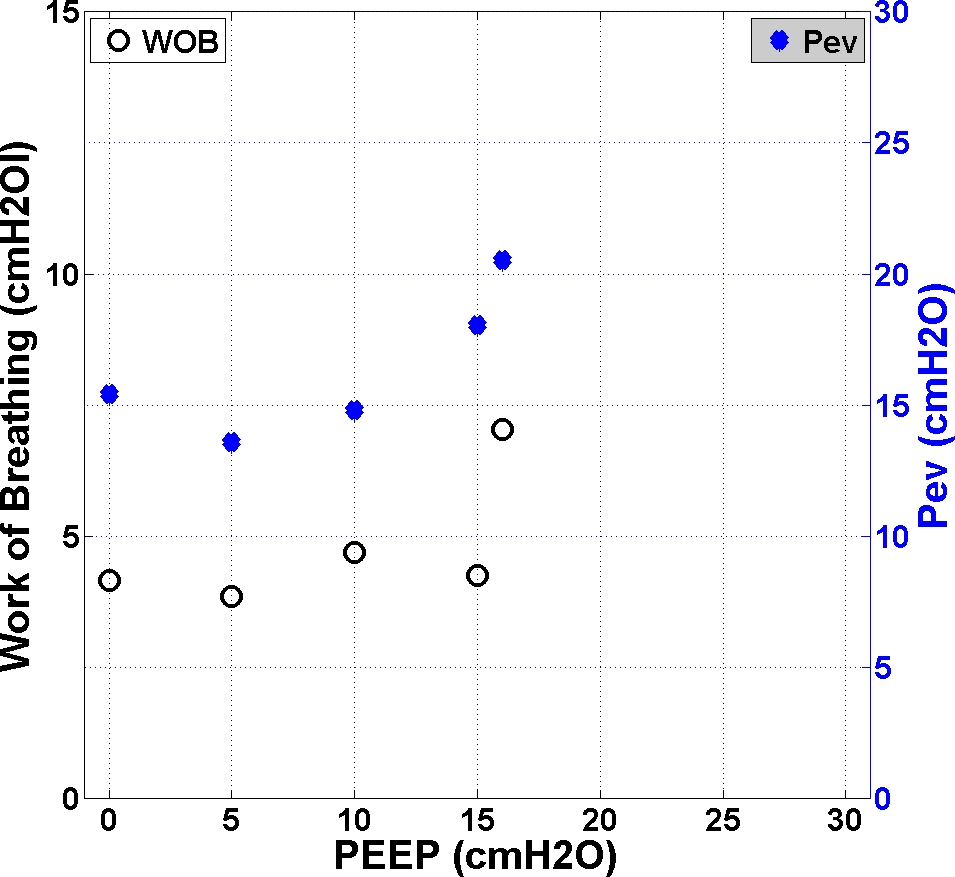 |
| **Patient 8** | |
| 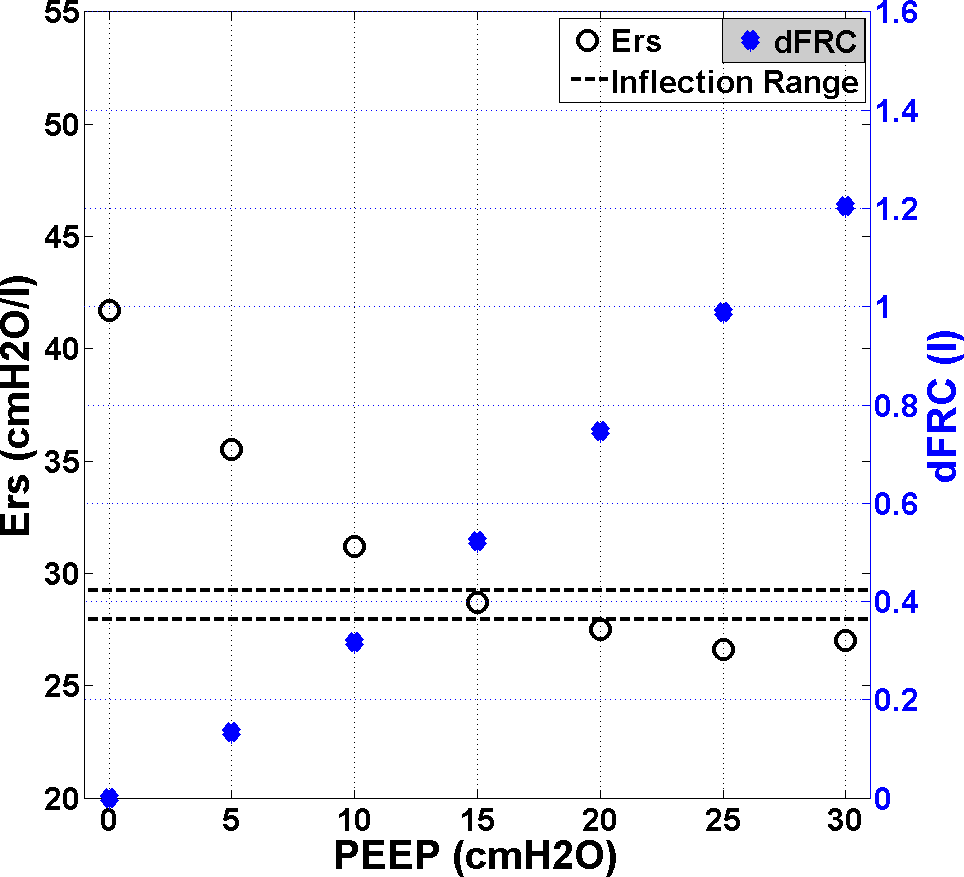 | 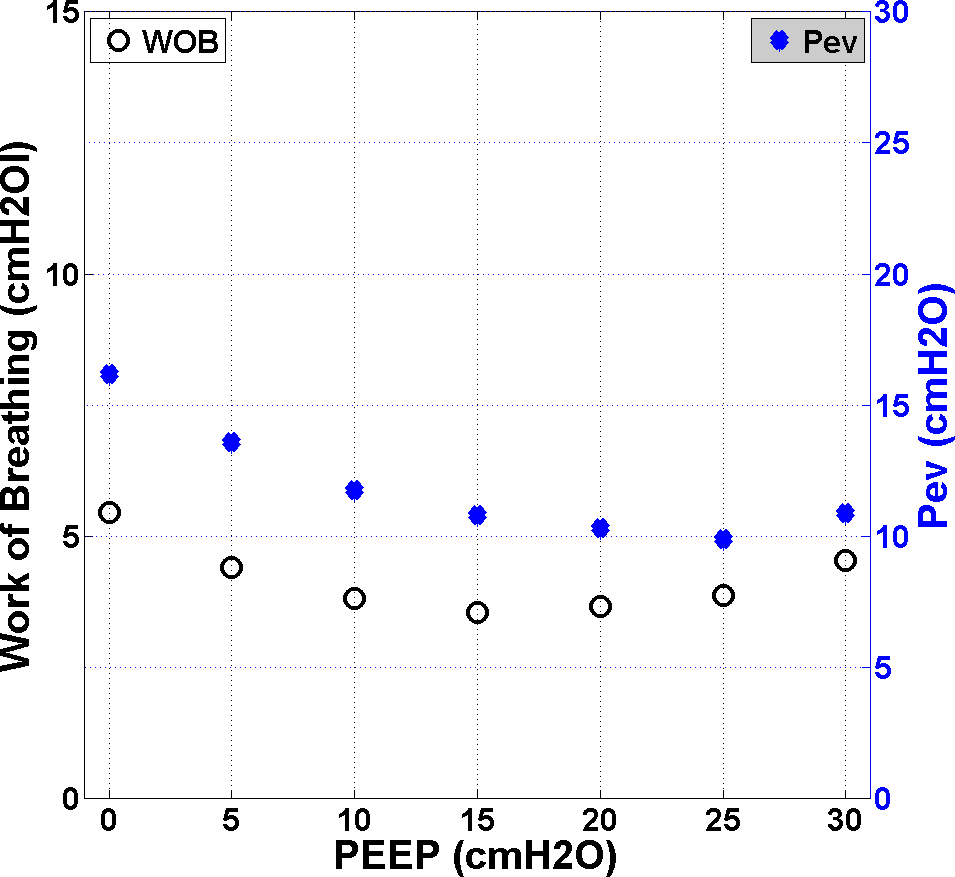 |
| **Patient 9** | |
| 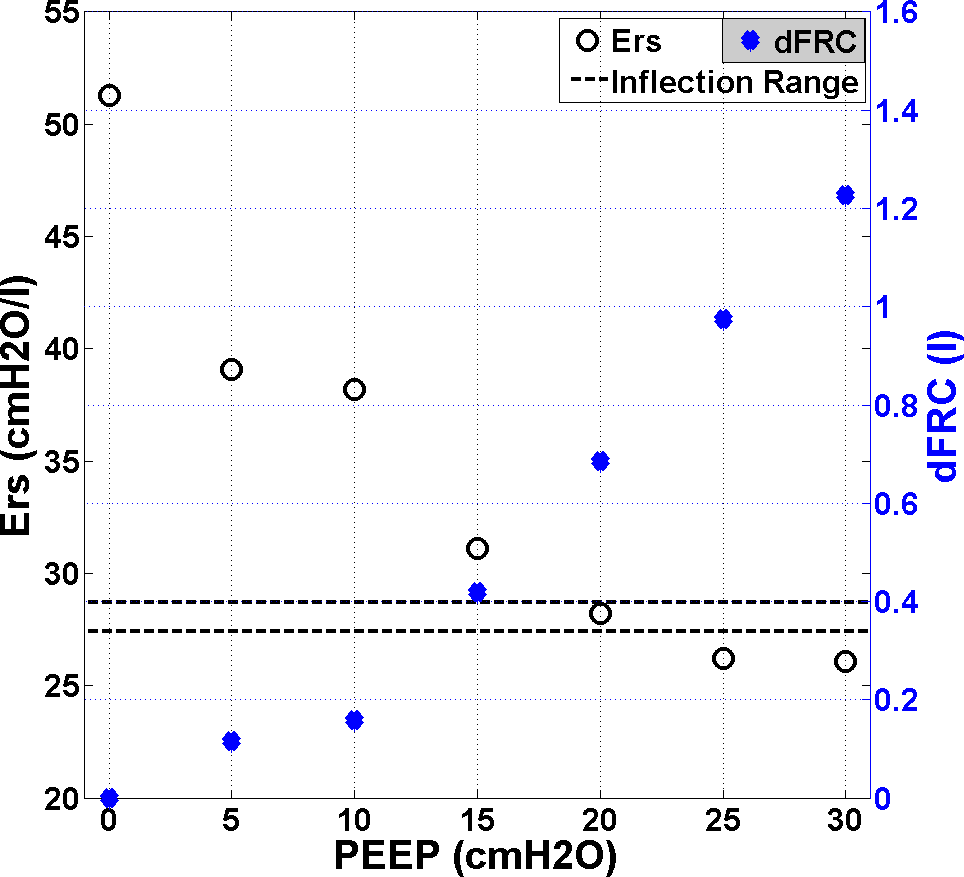 | 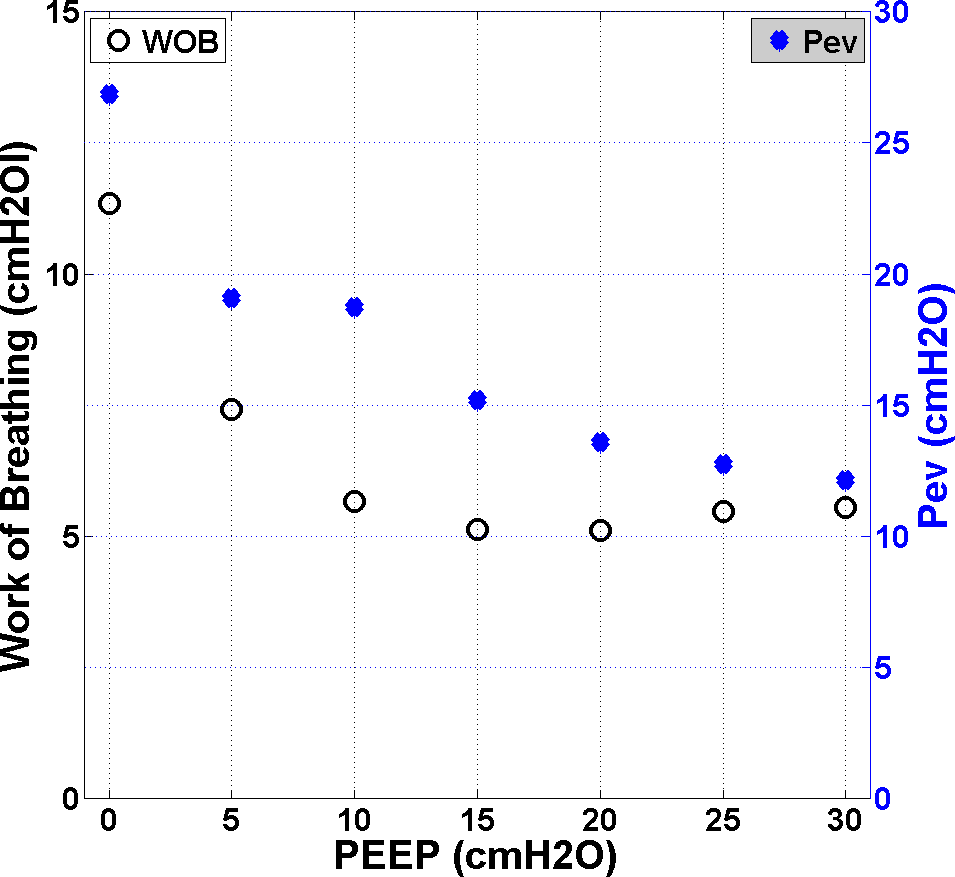 |
| **Patient 10** | |
| 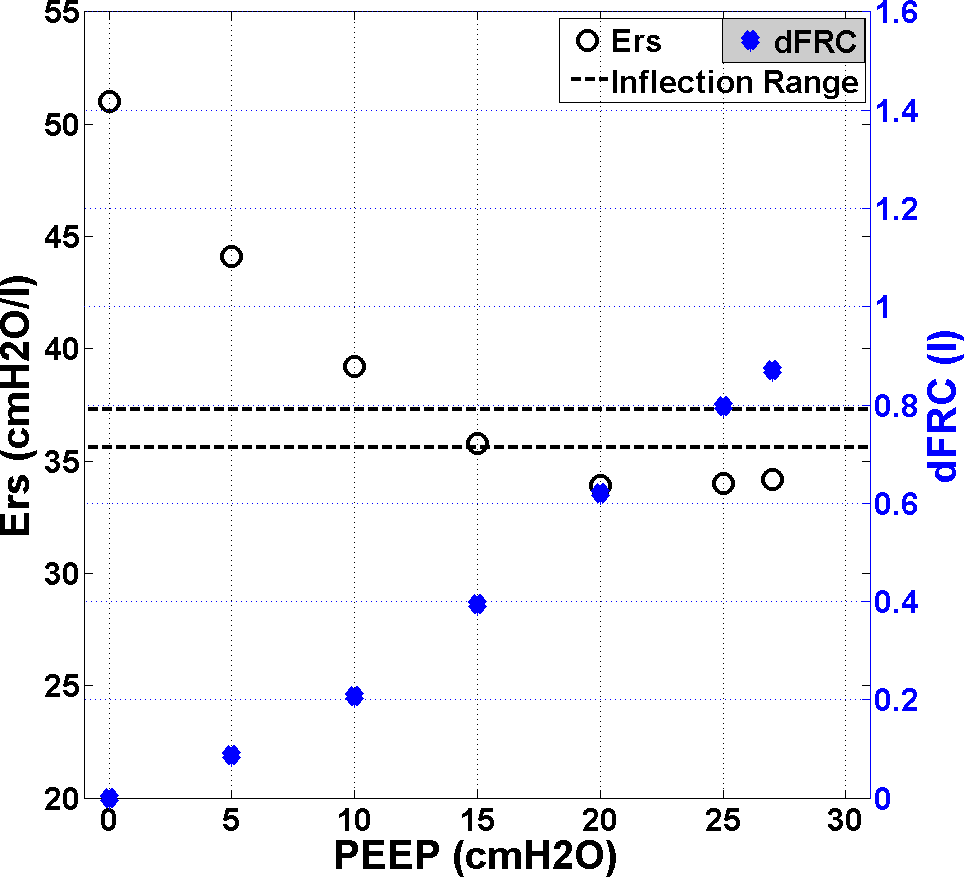 | 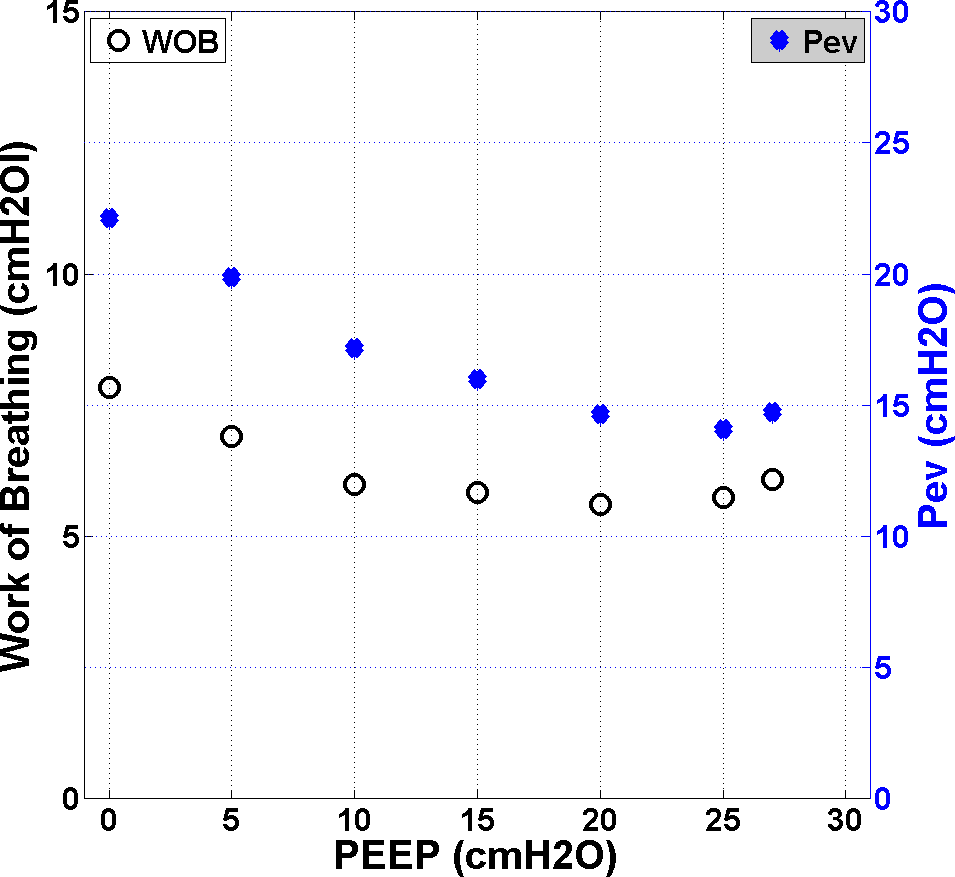 |

**3.0 Hemodynamic Stability:**

During recruitment manoeuvre, there was no standard usage of drugs or fluids to maintain patient hemodynamic tolerance towards increase of intrathoracic pressures.

There were several patients who were given isotonic crystalloid (*Plasma-lyte*) and/or *noradrenaline* prior or after the clinical protocol:

Patient 3 – 1 hour after the clinical protocol, the patient was given *Plasma-lyte*.

Patient 4 – 2 hours before the clinical protocol, patient was given *Plasma-lyte*.

Patient 6 – Patient was given *noradrenaline* as a part of an on-going treatment.

Patient 7 – Patient was given *noradrenaline* as a part of an on-going treatment.

Patient 10 – Patient was give *noradrenaline* as a part of an on-going treatment.

**4.0 Independent Patient Study – Patient 9 (H1N1)**

Patient 9 is an interesting case, where *E_rs_* only decreases by less than 1.0 *cmH2O/l* from PEEP of 5 to 10 *cmH_2_O*. However, when PEEP is increased to 15 *cmH_2_O*, *E_rs_* drops significantly, with a significant increase of dFRC, as shown in Figure E2. This result suggests that from PEEP of 5 to 10 *cmH_2_O*, minimal lung volume is recruited resulting in smaller *E_rs_* drop. The significant drop in *E_rs_* at PEEP 15 *cmH_2_O* indicates that PEEP = *15 cmH_2_O* has overcome resistance to recruitment and thus more new lung volume is recruited. The *E_rs_* drop with the lung volume increase at other PEEP levels was less significant. Patient 9 was admitted to ICU with H1N1 and high PEEP MV treatment to recruit the lung has proven to be beneficial for these patients . This specific case illustrates the potential of monitoring *E_rs_* to capture the unique patient-specific lung recruitment and condition as it occurs in a clinically and physiologically relevant manner. This specific case further suggested that the use minimal *E_rs_* PEEP is generalizable in a heterogeneous ARDS cohort.


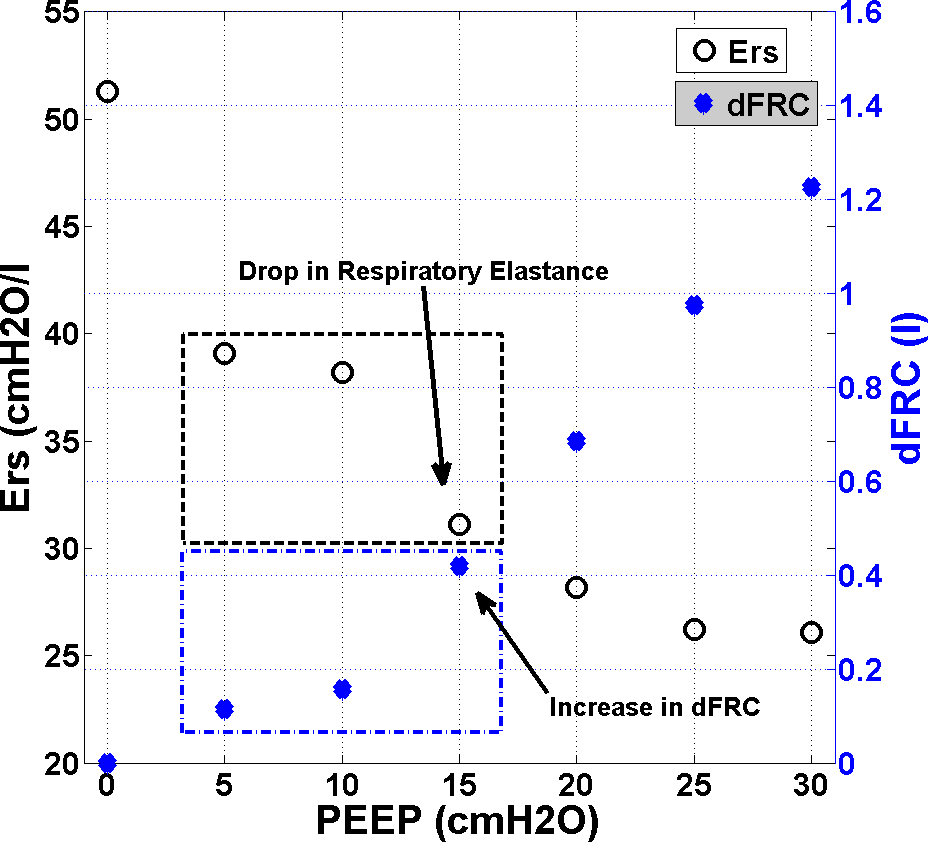


**Figure E2:** ***E_rs_*-PEEP and dFRC-PEEP curve for Patient 9.** The increase in dFRC result in *E_rs_* drop when PEEP is increased from 10 to 15 *cmH_2_O.*

**5.0 References:**

1. Lucangelo U, Bernabè F, Blanch L: **Lung mechanics at the bedside: make it simple.** *Current Opinion in Critical Care* 2007, **13:**64-72

2. Brochard L, Martin G, Blanch L, Pelosi P, Belda FJ, Jubran A, Gattinoni L, Mancebo J, Ranieri VM, Richard J-C, Gommers D, Vieillard-Baron A, Pesenti A, Jaber S, Stenqvist O, Vincent J-L: **Clinical review: Respiratory monitoring in the ICU - a consensus of 16.** *Critical Care* 2012, **16:**219.

3. Gattinoni L, Caironi P, Cressoni M, Chiumello D, Ranieri VM, Quintel M, Russo S, Patroniti N, Cornejo R, Bugedo G: **Lung Recruitment in Patients with the Acute Respiratory Distress Syndrome.** *N Engl J Med* 2006, **354:**1775-1786.

4. Barberis L, Manno E, Guérin C: **Effect of end-inspiratory pause duration on plateau pressure in mechanically ventilated patients.** *Intensive Care Medicine* 2003, **29:**130-134.

5. Briel M MMMA, et al.: **Higher vs lower positive end-expiratory pressure in patients with acute lung injury and acute respiratory distress syndrome: Systematic review and meta-analysis.** *JAMA: The Journal of the American Medical Association* 2010, **303:**865-873.

6. Ramsey CD, Funk D, Miller RRI, Kumar A: **Ventilator management for hypoxemic respiratory failure attributable to H1N1 novel swine origin influenza virus.** *Critical Care Medicine* 2010, **38:**e58-e65.
